# Spontaneous Cervical Epidural Hematoma Mimicking Crowned Dens Syndrome: A Case Report

**DOI:** 10.7759/cureus.93480

**Published:** 2025-09-29

**Authors:** Katsuyuki Shiose, Koki Tsuchiya, Ichiro Okano, Yoshifumi Kudo

**Affiliations:** 1 Department of Orthopaedic Surgery, SHOWA Medical University School of Medicine, Tokyo, JPN

**Keywords:** acute neck pain, anticoagulant therapy, crowned dens syndrome, odontoid calcification, spontaneous cervical epidural hematoma

## Abstract

Crowned dens syndrome (CDS) typically manifests as acute neck pain accompanied by calcifications around the odontoid process. However, such findings may obscure more serious underlying pathologies. We report a case of an 85-year-old man on edoxaban who presented with acute neck pain and atlantoaxial calcifications on computed tomography (CT), initially suggestive of CDS. Subsequent mild neurological deficits prompted cervical magnetic resonance imaging (MRI), which revealed a spontaneous cervical epidural hematoma (SCEH) extending from C2 to C4. The patient was managed conservatively and achieved complete recovery. This case highlights the importance of considering SCEH in the differential diagnosis of severe neck pain, even when odontoid calcification is observed on CT, especially in patients receiving anticoagulant therapy. Thorough clinical evaluation and close follow-up are essential for timely diagnosis and management.

## Introduction

Crowned dens syndrome (CDS) is a rare inflammatory condition caused by deposition of calcium pyrophosphate dihydrate or hydroxyapatite crystals around the odontoid process, resulting in severe acute neck pain and restricted cervical range of motion [1,2]. CDS primarily affects elderly individuals and frequently presents with systemic inflammatory signs such as fever, leukocytosis, and elevated C-reactive protein (CRP) [1]. The diagnosis is often confirmed by computed tomography (CT), which demonstrates characteristic “crown-like” calcifications surrounding the C1-C2 region. However, odontoid calcifications are not pathognomonic for CDS; asymptomatic periodontoid calcifications have been reported in up to 13.5% of patients with suspected brain disease undergoing cervical CT [3]. Therefore, the diagnosis based solely on imaging can be misleading. Previous reports have described cases in which meningitis and cervical epidural abscess mimicked CDS; however, spontaneous cervical epidural hematoma (SCEH) mimicking CDS has not been reported [4,5].

SCEH is a rare but potentially serious condition characterized by acute neck or back pain and neurological deficits, often associated with anticoagulant therapy or coagulopathies [6,7]. Magnetic resonance imaging (MRI) is essential for diagnosis, yet its use may be delayed or overlooked when alternative diagnoses such as CDS are favored. Herein, we report a rare case of SCEH initially misdiagnosed as CDS due to overlapping clinical features and misleading CT findings.

## Case presentation

**Figure 1:**
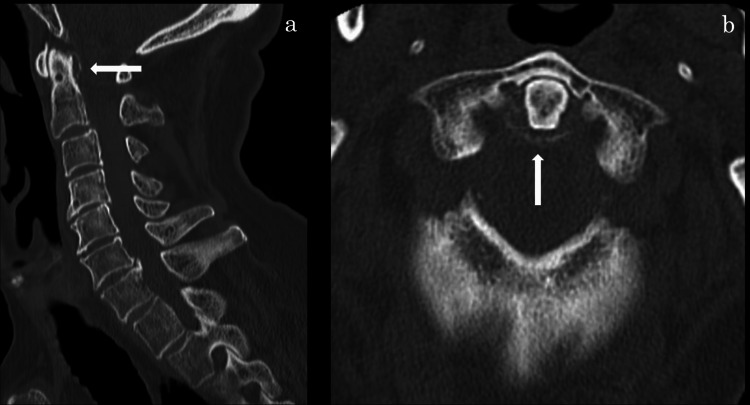
CT scan of the cervical spine

An 85-year-old man presented to our emergency department with acute, severe neck pain radiating to both shoulders. Cervical motion was markedly restricted, but no motor or sensory deficits were initially observed. His medical history was significant for atrial fibrillation and ischemic stroke; he was on edoxaban therapy. Cervical CT demonstrated calcification of the atlantoaxial joint (Figure 1), leading to an initial diagnosis of CDS.

**Figure 2:**
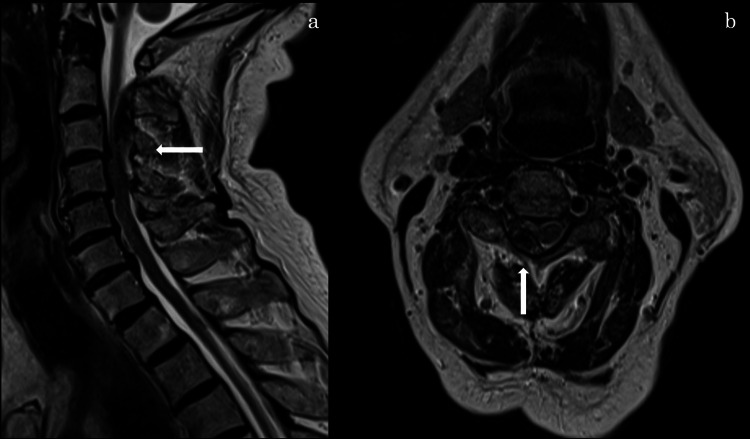
MRI T2-weighted image (a) T2-weighted sagittal image showing a hematoma located dorsally at the C2–C4 vertebral levels. (b) T2-weighted axial image showing an extradural hematoma compressing and displacing the spinal cord at the C2–C4 levels.

Conservative management was initiated. Two days later, the patient returned with persistent neck pain. Laboratory tests revealed mild leukocytosis, but CRP levels remained within normal limits. Manual muscle testing revealed mild weakness in the right deltoid and biceps brachii muscles. On day 7, cervical MRI revealed a right-sided posterior epidural hematoma extending from C2 to C4 with spinal cord compression (Figure 2), confirming the diagnosis of SCEH, and edoxaban therapy was subsequently discontinued.

**Figure 3:**
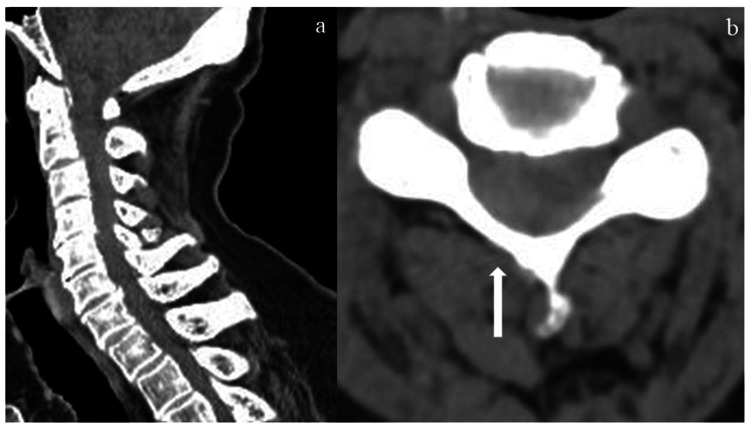
CT scan of cervical spine in a soft tissue window (a) Sagittal CT scan of the cervical spine. (b) Axial CT scan of the cervical spine revealed a right-sided shadow suggestive of an epidural hematoma.

Moreover, retrospective evaluation of the initial cervical CT in a soft tissue window revealed an indistinct but recognizable shadow suggestive of an epidural hematoma (Figure 3).

**Figure 4:**
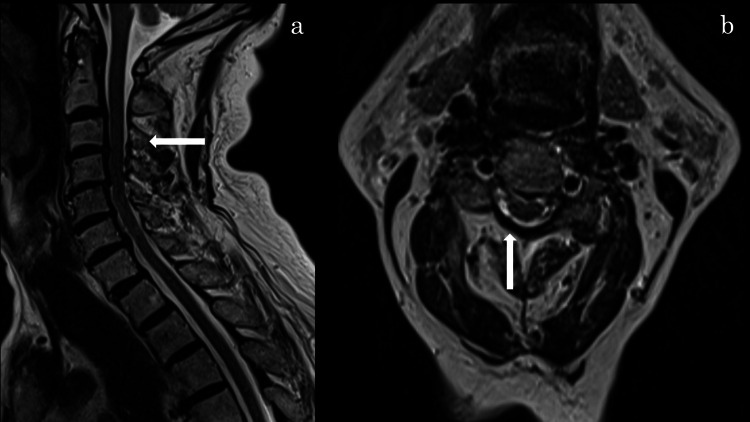
MRI T2-weighted images Sagittal (a) and axial (b) T2-weighted images showing changes following edoxaban discontinuation at the C2–C4 levels.

By day 9, the patient's neck pain and muscle strength had improved; he was managed conservatively. At day 26, pain had resolved and muscle strength fully recovered, and edoxaban therapy was resumed. Follow-up MRI at one month showed significant reduction of the hematoma without residual spinal cord compression (Figure 4).

The patient remained neurologically intact at his final follow-up.

## Discussion

CDS is an inflammatory disorder caused by calcium crystal deposition around the odontoid process, resulting in sudden, severe neck pain and cervical stiffness [1,2]. It primarily affects elderly patients, with CT typically revealing “crown-like” calcifications around the dens. Lu et al. reported that calcification of the odontoid process associated with CDS was most commonly identified at a mean age of 71 years, with a prevalence of 6% in individuals over 50 years of age, increasing to 20% among those aged 80-89 years [8]. However, incidental periodontoid calcifications are common among the elderly and can lead to diagnostic confusion if clinical correlation is lacking [3].

In our patient, although several clinical and radiological features initially suggested CDS, he lacked systemic inflammatory signs such as fever and elevated CRP. While normal inflammatory markers do not exclude CDS, they warrant caution in confirming the diagnosis [1,9].

SCEH, a rare but critical differential diagnosis, presents with acute neck pain and neurological deficits, frequently occurring in patients on anticoagulants or with coagulopathies [6,7,10]. Anticoagulant therapy and coagulopathy may predispose patients to spontaneous epidural hematoma by disrupting normal hemostasis.

The incidence of SCEH increases with age and may mimic other cervical pathologies such as CDS. In this case, the patient's anticoagulation therapy and new-onset neurological deficits raised suspicion for SCEH. MRI remains the gold standard for diagnosing epidural hematomas, showing characteristic signal changes on T1- and T2-weighted images [11]. Retrospective review of the soft tissue window CT suggested a hematoma; however, the diagnosis would have been difficult without considering it in the differential.

This case highlights several key considerations. First, periodontoid calcifications are not exclusive to CDS and can coexist with other serious, unrelated conditions, especially in elderly patients. Second, in patients presenting with acute neck pain who are receiving anticoagulation therapy, hemorrhagic causes should be carefully considered. In addition, even subtle neurological deficits warrant advanced imaging such as MRI to prevent delayed diagnosis and the risk of permanent spinal cord injury. Overall, this case underscores the diagnostic challenges posed by the overlapping clinical features of CDS and SCEH and emphasizes the need for thorough clinical evaluation beyond imaging findings, particularly in elderly or anticoagulated patients.

## Conclusions

In the present case, the initial CT suggested CDS, but due to anticoagulation therapy and mild neurological deficits, an MRI was performed and revealed SCEH. This case illustrates that SCEH can mimic CDS, especially in elderly patients with odontoid calcifications. Reliance solely on CT findings may delay diagnosis, particularly in those receiving anticoagulant therapy. Clinicians should consider SCEH when neurological symptoms or hemorrhagic risk factors are present. Careful clinical evaluation and close follow-up are essential in such cases.
